# Valine Pyrrolidide Preserves Intact Glucose-Dependent Insulinotropic Peptide and Improves Abnormal Glucose Tolerance in Minipigs With Reduced β-Cell Mass

**DOI:** 10.1155/EDR.2003.93

**Published:** 2003

**Authors:** Marianne Olholm Larsen, Bidda Rolin, Ulla Ribel, Michael Wilken, Carolyn F. Deacon, Ove Svendsen, Carsten F. Gotfredsen, Richard David Carr

**Affiliations:** 1 Department of Pharmacological Research I Novo Nordisk A/S Bagsvaerd Denmark; 2 Department of Pharmacology and Pathobiology the Royal Veterinary and Agricultural University Copenhagen Denmark; 3 Department of Assay and Cell Technology Novo Nordisk A/S Bagsvaerd Denmark; 4 Department of Medical Physiology The Panum Institute University of Copenhagen Copenhagen Denmark; 5 Department of Histology Novo Nordisk A/S Bagsvaerd Denmark; 6 Department of Pharmacological Research I, Pharmacology, Research and Development Novo Allé, 1Q1.28 Bagsvaerd DK-2880 Denmark

## Abstract

The incretin hormones glucagon-like peptide-1 (GLP-1) and glucose-dependent insulinotropic polypeptide (GIP) are important in blood glucose regulation.However, both incretin hormones are rapidly degraded by the enzyme dipeptidyl peptidase IV (DPPIV). The concept of DPPIV inhibition as a treatment for type 2 diabetes was evaluated in a new large animal model of insulin-deficient diabetes and reduced *β*-cell mass, the nicotinamide (NIA) (67 mg/kg) and streptozotocin (STZ) (125 mg/kg)–treated minipig, using the DPPIV inhibitor, valine pyrrolidide (VP) (50 mg/kg).VP did not significantly affect levels of intact GLP-1 but increased levels of intact GIP (from 4543 ± 1880 to 9208 ± 3267 pM × min; *P*<.01), thus improving glucose tolerance (area under the curve [AUC] for glucose reduced from 1904 ± 480 to 1582 ± 353 mM × min;*P* = .05).VP did not increase insulin levels during the oral glucose tolerance test (OGTT) but increased the insulinogenic index in normal animals (from 83 ± 42 to 192 ± 108; *P* < .05), but not after NIA + STZ, possibly because of less residual insulin secretory capacity in these animals. GIP seems to contribute to the antihyperglycemic effect of VP in this model; however, additional mechanisms for the effect of DPPIV inhibition cannot be excluded. The authors conclude that DPPIV inhibitors may be useful to treat type 2 diabetes, even when this is due to reduced *β*-cell mass.


*Experimental Diab. Res.*, 4:93–105, 2003
Copyright © Taylor and Francis Inc.
ISSN: 1543-8600 print / 1543-8619 online
DOI: 10.1080/15438600390220034

### Valine Pyrrolidide Preserves Intact Glucose-Dependent Insulinotropic Peptide and Improves Abnormal Glucose Tolerance in Minipigs With Reduced β-Cell Mass

**Marianne Olholm Larsen,[1,2] Bidda Rolin,[1] Ulla Ribel,[1] Michael Wilken,[3] Carolyn F. Deacon,[4] Ove Svendsen,[2] Carsten F. Gotfredsen,[5] and Richard David Carr[1]**

[1]*Department of Pharmacological Research I, Novo Nordisk A/S, Bagsvaerd, Denmark*
[2]*Department of Pharmacology and Pathobiology, the Royal Veterinary and Agricultural University, Copenhagen, Denmark*
[3]*Department of Assay and Cell Technology, Novo Nordisk A/S, Bagsvaerd, Denmark*
[4]*Department of Medical Physiology, The Panum Institute, University of Copenhagen, Copenhagen, Denmark*
[5]*Department of Histology, Novo Nordisk A/S, Bagsvaerd, Denmark*

**The incretin hormones glucagon-like peptide-1 (GLP-1) and glucose-dependent insulinotropic polypeptide (GIP) are important in blood glucose regulation. However, both incretin hormones are rapidly degraded by the enzyme dipeptidyl peptidase IV (DPPIV). The concept of DPPIV inhibition as a treatment for type 2 diabetes was evaluated in a new large animal model of insulin-deficient diabetes and reduced β-cell mass, the nicotinamide (NIA) (67 mg/kg) and streptozotocin (STZ) (125 mg/kg)–treated minipig, using the DPPIV inhibitor, valine pyrrolidide (VP) (50 mg/kg). VP did not significantly affect levels of intact GLP-1 but increased levels of intact GIP (from 4543 ± 1880 to 9208 ± 3267 pM × min; $P < .01$), thus improving glucose tolerance (area under the curve [AUC] for glucose reduced from 1904 ± 480 to 1582 ± 353 mM × min; $P = .05$). VP did not increase insulin levels during the oral glucose tolerance test (OGTT) but increased the insulinogenic index in normal animals (from 83 ± 42 to 192 ± 108; $P < .05$), but not after NIA + STZ, possibly because of less residual insulin secretory capacity in these animals. GIP seems to contribute to the antihyperglycemic effect of VP in this model; however, additional mechanisms for the effect of DPPIV inhibition cannot be excluded. The authors conclude that DPPIV inhibitors may be useful to treat type 2 diabetes, even when this is due to reduced β-cell mass.**

**Keywords** Dipeptidyl Peptidase; Glucose Tolerance; Insulin-Deficient Diabetes; In Vivo Pharmacology; Streptozotocin

The incretin hormones glucagon-like peptide-1 (GLP-1) and glucose-dependent insulinotropic polypeptide (GIP) enhance postprandial insulin secretion in a glucose-dependent manner [14, 53, 69]. GLP-1 increases levels of insulin mRNA, promotes insulin biosynthesis [16, 68], and inhibits glucagon secretion [53], although this may be secondary to GLP-1–induced somatostatin secretion [21]. GLP-1 also inhibits gastrointestinal motility and gastric acid secretion [70, 72], thereby limiting postprandial glucose excursions, although more recently, direct effects on appetite regulation have been reported [17, 45]. GIP has been indicated to promote glucose uptake in muscle [51] and fat [52] and has been suggested to increase the affinity of the insulin receptor [61]. These observations gave rise to the suggestion that GIP may modulate the effects of insulin by

Received 14 November 2002; accepted 16 March 2003.
Part of this study was supported by a grant from the Novo Nordisk Foundation. The authors thank Helle Nygaard, Margit Jeppesen, Birgitte Roed, Merete Hvidt, Anne-Grethe Juul, Lotte Gottlieb Sørensen, Nanna Kasmira Nowa Hansen, Annemette Petersen, Karsten Larsen, and Hans Rasmussen for excellent technical assistance.
Address correspondence to Marianne O. Larsen, Department of Pharmacological Research I, Pharmacology, Research and Development, Novo Allé, 1Q1.28, DK-2880 Bagsvaerd, Denmark. E-mail: mmla@novonordisk.com directly altering target tissue sensitivity to insulin [52], thereby contributing to the effective uptake of glucose postprandially [44, 73]. Receptor antagonists have been used to demonstrate the physiological role of both incretin hormones in the maintenance of normal glucose homeostasis [13, 38, 59, 66]; mice with targeted deletions of either the GLP-1 or the GIP receptor become glucose intolerant [43, 60], confirming the importance of both peptides in the regulation of blood glucose.

The insulinotropic effect of GLP-1 is preserved in patients with type 2 diabetes [14, 22, 46, 47, 65], as are its effects on glucagon secretion [48] and gastric emptying [71], whereas it has been suggested that GIP loses its effect in these subjects [14, 47], possibly because of a defect in the expression of the GIP receptor [25]. GLP-1 can normalize glucose levels in type 2 diabetic patients [49] and restore insulin secretion towards normal in impaired glucose tolerant subjects [18], meaning that the peptide could have considerable therapeutic application.

However, both GLP-1 and GIP are rapidly degraded to apparently inactive metabolites by the enzyme dipeptidyl peptidase IV (DPPIV) [10, 11, 30, 41], although GIP seems less susceptible to degradation than GLP-1 [9, 10]. Inhibition of DPPIV is known to protect the biologically active forms of GLP-1 and GIP from N-terminal breakdown [7, 8], and is a newly emerging principle for drug treatment of type 2 diabetes [11, 24]. The rationale for this principle is that by protecting the endogenous intact forms of the incretin hormones, their beneficial effects on glucose tolerance will be enhanced. The approach of enhancing levels of intact endogenous incretin hormones by inhibition of DPPIV has been effective in improving glucose tolerance in several animal models [1, 4, 56, 57, 63] and has recently been shown to improve metabolic control in type 2 diabetic humans [2]. However, because the principle of inhibiting DPPIV relies on an improved incretin effect, this might require that functional $\beta$-cell mass has a sufficient capacity to respond to the insulinotropic effect of increased incretin levels before an effect on metabolic control is seen. The animal studies used insulin-resistant rodents [1, 4, 56, 57, 63], whereas the study in human subjects included mildly diabetic subjects (fasting blood glucose $<10$ mM) [2]. Whether DPPIV inhibition will be effective in subjects with reduced $\beta$-cell mass remains to be determined. The aim of the present study was, therefore, to evaluate the concept of DPPIV inhibition for treatment of reduced glucose tolerance due to a reduction of $\beta$-cell mass. The model used was a new large-animal model of mild insulin-deficient diabetes, the nicotinamide (NIA) and streptozotocin (STZ)–treated Göttingen minipig [37]. The Göttingen minipig was chosen because its size makes it possible to obtain a relatively large number of blood samples, thus making this species useful in the study of GLP-1 and GIP metabolism. Furthermore, the pig shares many anatomical and physiological similarities of digestion and metabolism with the human [6, 42, 64] and the Göttingen minipig has been characterized in detail, both with respect to general characteristics such as clinical chemistry and hematology [15, 19, 29] and, more specifically, with respect to glucose and lipid metabolism both in normal animals [28, 35, 36, 37] and after induction of diabetes [31, 37] or challenge with high-fat diets [26–28, 36].

### MATERIALS AND METHODS

### Animals

Six male Göttingen minipigs, obtained from the barrier unit at Ellegaard Göttingen minipigs ApS, Denmark, 11 to 14 months of age and weighing 25.3 ± 1.4 kg (range 22.8 to 26.8 kg) were used. The animals were housed in single pens under controlled conditions (temperature was kept between 18°C and 22°C, relative air humidity was 30% to 70%, with 4 air changes per hour), with a 12-hour light:12-hour dark cycle. The animals were fed a restricted diet, 140 g of SDS minipig diet (SDS, Essex, England) and 240 g of a commercial swine fodder ("Svinefoder 22", Slangerup, Denmark), twice daily and were allowed free access to water. The pigs were trained carefully in all experimental procedures before start of experiments. Animals served as their own control and were thus studied both before and after induction of diabetes, with and without dosing of valine pyrrolidide (VP), a specific DPPIV inhibitor [50].

The type of study was approved by the Animal Experiments Inspectorate, Ministry of Justice, Denmark.

### Surgical Implantation of Central Venous Catheters

Two central venous catheters (Certo 455, B. Braun Melsungen AG, Melsungen, Germany) were inserted surgically under general anesthesia as described previously [37]. Postsurgical analgesia was maintained by injection of buprenorfine, 0.03 mg/kg (Anorfin (0.3 mg/mL); GEA, Frederiksberg, Denmark) and carprofen, 4 mg/kg (Rimadyl vet. (50 mg/mL); Pfizer, Ballerup, Denmark) intramuscularly before the end of anesthesia and for 3 days post surgery by injection of carporfen, 4 mg/kg once daily intramuscularly. At the start of the study period, all animals had recovered fully from the surgical procedure as evaluated by normal behavior and eating patterns.

### Mixed-Meal Oral Glucose Tolerance Test

The mixed-meal oral glucose tolerance test (OGTT) was performed in all animals 1 and 2 weeks before exposure to NIA and STZ and was repeated 2 and 3 weeks after NIA + STZ. The test was performed in nonrestrained, freely moving animals in their home pens to reduce the amount of stress experienced by the animals during testing.

After an 18-hour overnight fast, animals were dosed intravenously with either vehicle (sterile saline (0.9%); SAD, Copenhagen, Denmark) or VP (50 mg/kg) 15 minutes in advance of being offered an OGTT of 25 g SDS minipig fodder and 2 g/kg glucose (500 g/L; SAD). The meal was eaten from a bowl under supervision. On average it took the pigs 2.5 ± 1.5 minutes (range 1 to 7.5 minutes) to eat the test meal.

Blood samples were obtained from the jugular vein catheters at $t = -30, -20, -1, 7.5$ (n = 3), 15, 30, 45, 60, 90, 120, and 240 minutes relative to the beginning of ingestion of the glucose load.

### Handling and Analysis of Blood Samples

Three milliliters of full blood was obtained per sample and was immediately transferred to vials containing EDTA (1.6 mg/mL, final concentration), aprotinin 500 kIU/mL full blood (Trasylol, 10,000 kIU/mL; Bayer, Lyngby, Denmark) and VP (0.01 mM, final concentration, except for plasma for determination of DPPIV activity, where VP was not added) and kept on ice until centrifugation. Samples were centrifuged (4°C, 10 minutes, 3500 rpm), plasma separated, and stored at −20°C until analysis. Plasma glucose was analyzed by the hexokinase method using a Cobas Mira plus autoanalyzer (Roche Diagnostic Systems, Basel, Switzerland) following manufacturer's instructions. Plasma insulin was analyzed in a 2-site immunometric assay with monoclonal antibodies as catching and detecting antibodies (catching antibody HUI-018 raised against the A-chain of human insulin, detecting antibody OXI-005 raised against the B-chain of bovine insulin) [3] and using purified porcine insulin for calibration of the assay. The minimal detectable concentration was 3.2 pM and the upper limit was 1200 pM (no sample dilution). Inter- and intra-assay variations at 3 concentration levels were, respectively, 15.3% and 3.2% (at 342 pM), 9.9% and 7.6% (at 235 pM), and 14.6% and 4.4% (at 87 pM). Recovery at high-, medium-, and low-concentration levels was 97.1%, 97.9%, and 101%, respectively. A commercial kit from Linco was used to measure plasma glucagon (Glucagon RIA kit, catalogue number GL-32K) concentrations.

Plasma levels of intact and total incretin hormones were measured using specific radioimmunoassays that were validated by high-performance liquid chromatography (HPLC) as described previously [9, 10]. Total GIP was measured using the C-terminally directed antiserum R65 [33, 34]. The assay has a detection limit of <2 pM and an intra-assay variation of approximately 6%. Intact, biologically active GIP was measured using a newly developed assay [10]. The assay has a detection limit of approximately 5 pM, an $ED_{50}$ of 48 pM, and an intra-assay variation of <6%. VP (0.01 mM final concentration) was added to the assay buffer to prevent N-terminal degradation of GIP during the assay incubation. For both assays, human GIP (Peninsula Laboratories Europe, St Helens, Merseyside, UK) was used as standard (porcine GIP cross-reacts fully in both assays) and radiolabeled GIP was from Amersham Pharmacia Biotech (Little Chalfont, Buckinghamshire, UK).

GLP-1 concentrations were determined using assays directed toward each end of the molecule using human GLP-1 standards. N-terminal immunoreactivity was measured using a newly developed assay for the intact N-terminus of GLP-1 [12]. The assay has a detection limit of approximately 5 pM and an $ED_{50}$ of 80.8 ± 3.4 pM. The assay is specific for the intact N-terminus of GLP-1, and cross-reacts <0.1% with GLP-1(9–36) amide, or with the structurally related peptides GLP-1(1–36) amide, GIP(1–42), GIP(3–42), GLP-2(1–33), GLP-2(3–33), or glucagon at concentrations of up to 100 nM. It has a cross-reactivity of approximately 4% with the major proglucagon fragment (proglucagon(72–158)) secreted from the pancreas. Intra-assay variation was <6% and interassay variations were approximately 8% and 12% for 20 and 80 pM standards, respectively. VP (0.01 mM final concentration) was added to the assay buffer to prevent N-terminal degradation of GLP-1 during the assay incubation. HPLC supports the use of radioimmunoassays with this specificity for determination of intact GLP-1 [9]. Because both amidated and glycine-extended forms of GLP-1 are present in the pig [23], C-terminal immunoreactivity was determined using 2 different antisera (plasma samples for analysis of the glycine-extended form of GLP-1 were only available from 3 animals). Antiserum 89390 [75] has an absolute requirement for the intact amidated C-terminus of GLP-1(7–36) amide, and cross-reacts 83% with GLP-1(9–36) amide, but <0.01% with the glycine-extended form, GLP-1(7–37), or with C-terminally truncated fragments. Antiserum 92071 [75] is specific for the C-terminus of GLP-1(7–37) and cross-reacts fully with GLP-1(9–37) but <0.1% with amidated forms of GLP-1. For all assays, the intra-assay coefficient of variation was <6%. Plasma samples were extracted with 70% ethanol (vol/vol, final concentration) before assay, giving recoveries of 75% for GLP-1 added to plasma before extraction [54].

The activity of DPPIV in plasma samples was estimated by the ability to degrade GLP-1(7–37) added to the sample. The method is based upon the fact that DPPIV is the sole enzyme responsible for N-terminal degradation of GLP-1 in plasma [9, 55]. In brief, GLP-1(7–37) (5 $\mu$L, 100 fmol) was added to plasma samples (95 $\mu$L), which were then incubated for 1 hour at 37°C. Samples were put on an ice bath immediately after incubation and the amount of GLP-1 determined by an enzyme-linked immunosorbent assay (ELISA) specific for N-terminally intact peptide. A reference sample with GLP-1 added to heat-inactivated plasma containing VP (0.01 mM)

and aprotinin (500 kIU/mL) was processed in parallel. The DPPIV activity estimate was then calculated as

$$\text{Activity (\%)} = (1 - C_{\text{unknown}}/C_{\text{reference}}) \times 100$$

where $C_{\text{unknown}}$ and $C_{\text{reference}}$ are the calculated concentrations of the sample and the reference, respectively. The minimum detectable difference was determined to be 3.2%.

### $\beta$-Cell Reduction

$\beta$-Cell mass was reduced by intravenous administration of a combination of NIA (Sigma N-3376), 67 mg/kg, and STZ (Sigma S-0130), 125 mg/kg [37]. Administration of NIA and STZ was performed after an 18-hour overnight fast in conscious animals, and animals were offered SDS fodder 2 hours after treatment. Animals were observed frequently during the first 48 hours after administration of NIA and STZ and blood glucose was monitored regularly to avoid episodes of hypoglycemia due to sudden hyperinsulinemia caused by necrosis of $\beta$ cells.

### Histological Examination of Pancreas

*Fixation and Physical Fractionation*

After euthanasia with pentobarbitone (20 mL per animal) (200 mg/mL; Pharmacy of the Royal Veterinary and Agricultural University, Copenhagen, Denmark) at the end of the study period, the pancreas was isolated in toto for histological examination as previously described [37]. Histological examination was performed 41 $\pm$ 21 days (range 21 to 60) after NIA + STZ dosing in all animals and compared with data from normal, age-matched animals (n = 5). In short, pancreata were fixed in paraformaldehyde (Bie & Berntsen, Copenhagen, Denmark), embedded in 3% agar solution (catalogue number 303289, Meco-Benzon, Copenhagen, Denmark), and sectioned as practiced in the smooth fractionator method [5, 40]. The deparaffinized sections were stained for insulin and a mixture of antibodies to glucagon, somatostatin, and pancreatic polypeptide to visualize $\beta$ and non-$\beta$ endocrine cells.

Furthermore, sections were counterstained with Mayer's hematoxyline. Mass of $\beta$ and non-$\beta$ endocrine cells was evaluated stereologically in 2 to 3 sections with the origin of the sections blinded to the observer. Mass of endocrine cells is expressed at mg/kg body weight.

### Formulation of Compounds

VP (purity >98%) was synthesized by Dr. L. Christiansen, Novo Nordisk A/S, and was dissolved in physiological saline less than 60 minutes prior to administration. NIA was weighed out in individual portions and protected from light, and was dissolved in sterile saline (0.9%; SAD) to a concentration of 300 mg/mL immediately before injection. STZ was weighed out in individual portions and dissolved in sodium citrate buffer (catalogue number 929546), Bie & Berntsen), pH = 4.7, to a concentration of 62.5 mg/mL immediately before injection.

### Evaluation

Effects of VP on glucose tolerance and hormone levels were evaluated based on changes in postprandial plasma concentrations of glucose, insulin, and glucagon, as well as intact and total GLP-1 and GIP. Data are presented as mean $\pm$ SD.

The area under curve (AUC) for total and intact forms of the GLP-1 and GIP was calculated using the trapezoidal method (baseline = 0). For each incretin hormone, the percentage of intact peptide was expressed as $\text{AUC}_{\text{N-terminal}}$ relative to $\text{AUC}_{\text{C-terminal}}$. The glucose excursion and amounts of insulin secreted in response to the oral glucose load are expressed as incremental AUC, calculated after subtraction of the basal concentrations measured in samples taken before the glucose load. The insulinogenic index was calculated by dividing the incremental AUC for insulin by the incremental AUC for glucose during the postprandial period (0 to 240 minutes). All calculations and statistical evaluation of results were performed using paired 2-tailed Student's $t$ test and 1-way analysis of variance (ANOVA) using Excel (2000) and GraphPad Prism version 3.00 for Windows (GraphPad Software, San Diego, California, USA). $P$ values of .05 or less were considered significant.

### RESULTS

### Effects of NIA and STZ

Administration of NIA and STZ induced a significant increase in fasting plasma glucose without significant changes in fasting plasma values of insulin (Table 1). Furthermore, there were no significant changes in fasting plasma levels of any of the measured forms of GLP-1 and GIP or plasma DPPIV activity.

Administration of NIA and STZ induced a significant increase in $\text{AUC}_{\text{glucose}}$ after the OGTT, but no significant changes were seen in $\text{AUC}_{\text{insulin}}$ (Table 2). However, although $\text{AUC}_{\text{insulin}}$ was not significantly decreased, the insulin response after NIA + STZ was somewhat delayed and diminished compared to normal animals (Figure 1), with the ratio of insulin to glucose at 30 minutes being significantly reduced after NIA + STZ (22 $\pm$ 3 versus 51 $\pm$ 23; $P < .02$). Neither the absolute incretin concentrations nor the proportions of intact relative to total forms of GLP-1 and GIP were altered by NIA + STZ treatment (Table 3) (Figures 2 and 3), and DPPIV activity was not affected (Figure 4) (data on DPPIV activity are normalized according to baseline values in Figure 4 in order to illustrate the change in individual animals better).

**TABLE 1**
Fasting level of parameters before and after dosing of NIA (67 mg/kg) and STZ (125 mg/kg) in male Göttingen minipigs

| | Plasma glucose (mM) | Plasma insulin (pM) | N-terminal GLP-1 (pM) | C-terminal amidated GLP-1 (pM) | C-terminal glycine-extended GLP-1 (pM) | N-terminal GIP (pM) | C-terminal GIP (pM) |
|---|---|---|---|---|---|---|---|
| Before dosing | 3.6 ± 0.2 | 36 ± 6 | 12.3 ± 8.0 | 4.3 ± 3.2 | 9.9 ± 1.0 | 4.3 ± 1.8 | 7.6 ± 4.8 |
| After dosing | 4.8 ± 1.3 | 41 ± 21 | 13.1 ± 8.4 | 2.8 ± 1.7 | 8.6 ± 4.0 | 5.9 ± 2.4 | 17.1 ± 14 |
| *P* value | .05 | .45 | .80 | .43 | .68 | .14 | .08 |

*Note.* Data are presented as mean ± SD of individual plasma values (n = 6 for all parameters except C-terminal glycine-extended GLP-1, where n = 3). *P* values are based on paired Student's *t* test.

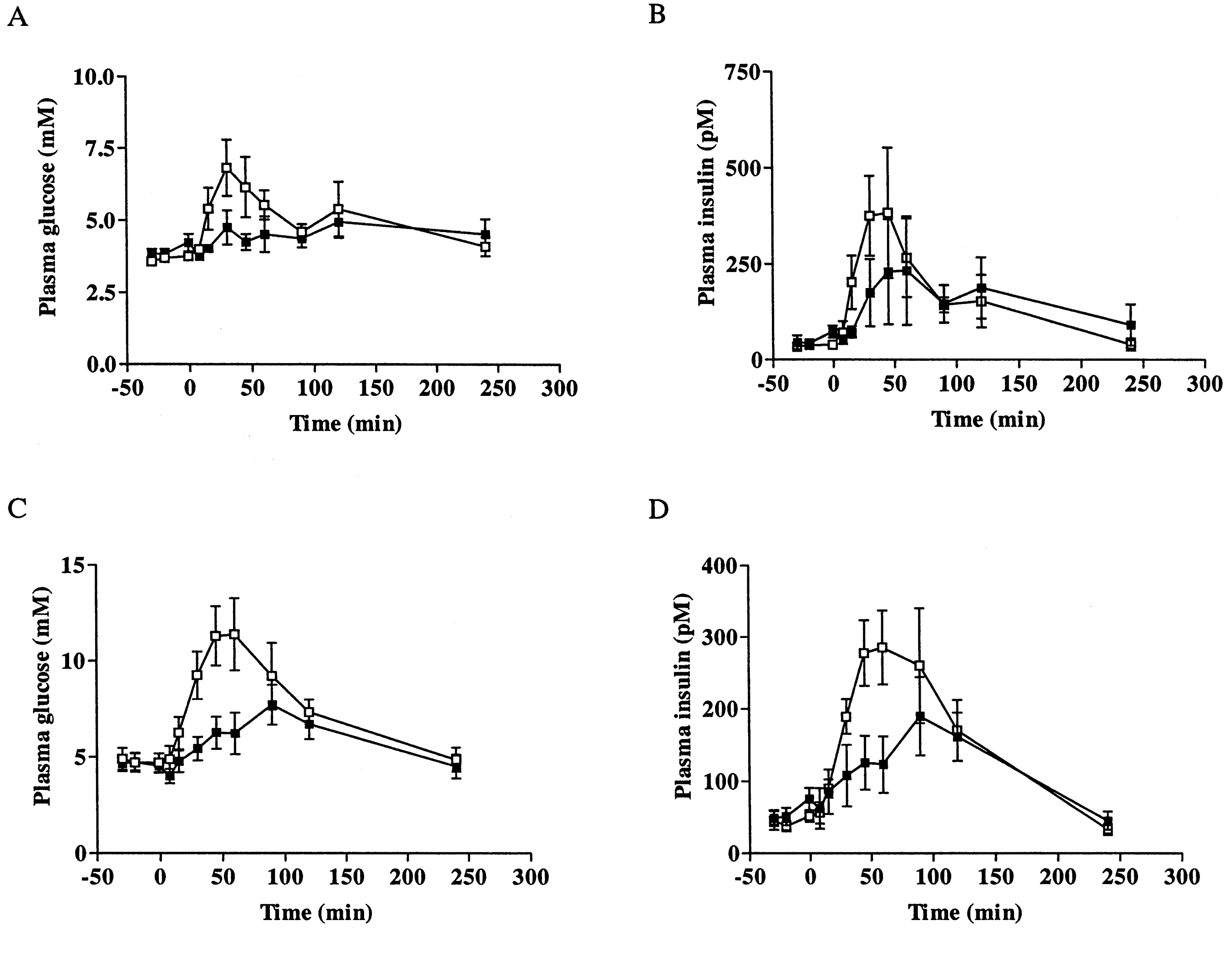

**FIGURE 1**
Plasma levels of glucose (*A*, *C*) and insulin (*B*, *D*) during OGTT in male Göttingen minipigs before (*A*, *B*) and after (*C*, *D*) dosing of NIA (6 mg/kg) and STZ (125 mg/kg). Animals are either dosed with VP (50 mg/kg) (■) or vehicle (□). Glucose (2 g/kg) ingested at $t = 0$ minutes, VP or vehicle dosed IV at $t = -15$ minutes; n = 6. Note different scales on Y-axis. Data are mean ± SEM.

**TABLE 2**
Values of incremental AUC for glucose and insulin during OGTT in male Göttingen minipigs

| Animals, dose[a] | Glucose (mM × min) | Insulin (pM × min) |
|---|---|---|
| Pre, Veh | 364 ± 295 | 30997 ± 26838 |
| Pre, VP | 194 ± 129 | 26950 ± 15010 |
| Post, Veh | 677 ± 339 | 27877 ± 14963 |
| Post, VP | 352 ± 179 | 16875 ± 8050 |
| *P* (Post, Veh versus Pre, Veh) | .05 | .64 |
| *P* (Pre, Veh versus Pre, VP) | .08 | .78 |
| *P* (Post, Veh versus Post, VP) | .02 | .09 |

*Note.* Data are presented as mean ± SD of individual values (n = 6 for all parameters). *P* values are based on paired Student's *t* test.
[a]Pre = normal animals; Post = animals dosed with NIA (67 mg/kg) + STZ (125 mg/kg); Veh = vehicle.

Based on stereological evaluation, $\beta$-cell mass (mg/kg) (7.02 ± 3.46 versus 17.68 ± 4.67 in normal animals [37] ($P < .001$)) was significantly reduced in animals dosed with NIA + STZ, whereas non-$\beta$-cell mass (mg/kg) was not changed (5.59 ± 0.86 versus 4.79 ± 1.30 in normal animals, nonsignificant [NS]). The pancreatic islets of animals dosed with NIA + STZ were found to be highly irregular due to disappearance of a large proportion of the $\beta$ cells. Furthermore, there was an apparent increase in small clusters of islet cells and of single $\beta$ and non-$\beta$ cells. No other abnormalities were discovered during morphological examination of pancreatic tissues.

### Effect of VP Administration

Plasma DPPIV activity was significantly reduced by VP both in normal animals and after NIA + STZ (Table 3, Figure 4);

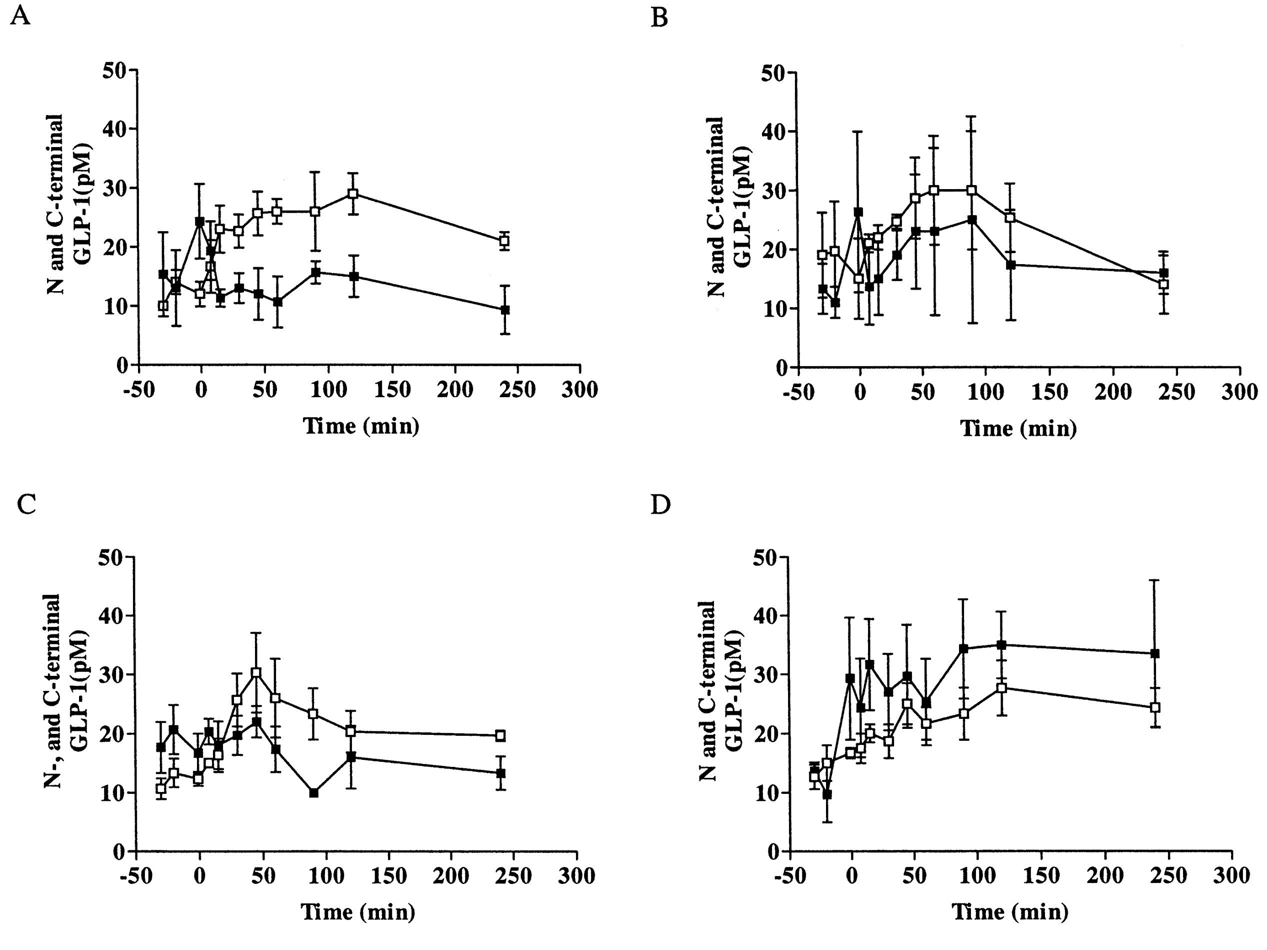

**FIGURE 2**
Plasma levels of intact (N-terminal) (■) and total (C-terminal) (□) GLP-1 during OGTT in male Göttingen minipigs before (*A*, *B*) and after (*C*, *D*) dosing of NIA (67 mg/kg) and STZ (125 mg/kg). Animals are dosed with either vehicle (*A*, *C*) or VP (50 mg/kg) (*B*, *D*). C-terminal GLP-1 includes both amidated and glycine-extended forms of the peptide. Glucose (2 g/kg) ingested at $t = 0$ minutes, VP or vehicle dosed IV at $t = -15$ minutes. Data are presented as mean ± SEM, n = 3.

**TABLE 3**
Values of AUC during OGTT in male Göttingen minipigs

| Animals, dose[a] | N-terminal GLP-1 (pM × min) | C-terminal amidated GLP-1 (pM × min) | Total C-terminal GLP-1 (pM × min) | N-terminal GIP (pM × min) | C-terminal GIP (pM × min) | N-/C-terminal (amidated + glycine-extended) GLP-1 (%) | N-/C-terminal (amidated + glycine-extended) GIP (%) | DPPIV (% × min) |
|---|---|---|---|---|---|---|---|---|
| Pre, Veh | 2215 ± 1608 | 2152 ± 558 | 6290 ± 1126 | 3836 ± 1337 | 22230 ± 10210 | 59 ± 12 | 18 ± 2 | 13410 ± 6384 |
| Pre, VP | 3723 ± 2843 | 2304 ± 1023 | 6117 ± 2602 | 10730 ± 4350 | 21420 ± 11090 | 75 ± 24 | 54 ± 12 | 4407 ± 7037 |
| Post, Veh | 2374 ± 2119 | 1730 ± 713 | 5550 ± 1029 | 4573 ± 1886 | 31100 ± 17410 | 80 ± 18 | 16 ± 4 | 14400 ± 3702 |
| Post, VP | 3778 ± 4145 | 1708 ± 956 | 6255 ± 1134 | 8808 ± 3481 | 17690 ± 8659 | 107 ± 36 | 53 ± 8 | 1494 ± 1368 |
| *P* (Post, Veh versus Pre, Veh) | .60 | .19 | .34 | .33 | .17 | .32 | .24 | .60 |
| *P* (Pre, Veh versus Pre, VP) | .18 | .81 | .94 | .02 | .91 | .15 | .001 | .01 |
| *P* (Post, Veh versus Post, VP) | .28 | .93 | .51 | .007 | .02 | .45 | .001 | .001 |

*Note*. Data are presented as mean ± SD of individual values (n = 6). *P* values are based on paired Student's *t* test. Values of AUC for total C-terminal GLP-1 and N-/C-terminal GLP-1 are based on the 3 animals where data for total C-terminal (amidated + glycine extended) GLP-1 were available.

[a]Pre = normal animals; Post = animals dosed with NIA (67 mg/kg) + STZ (125 mg/kg); Veh = vehicle.

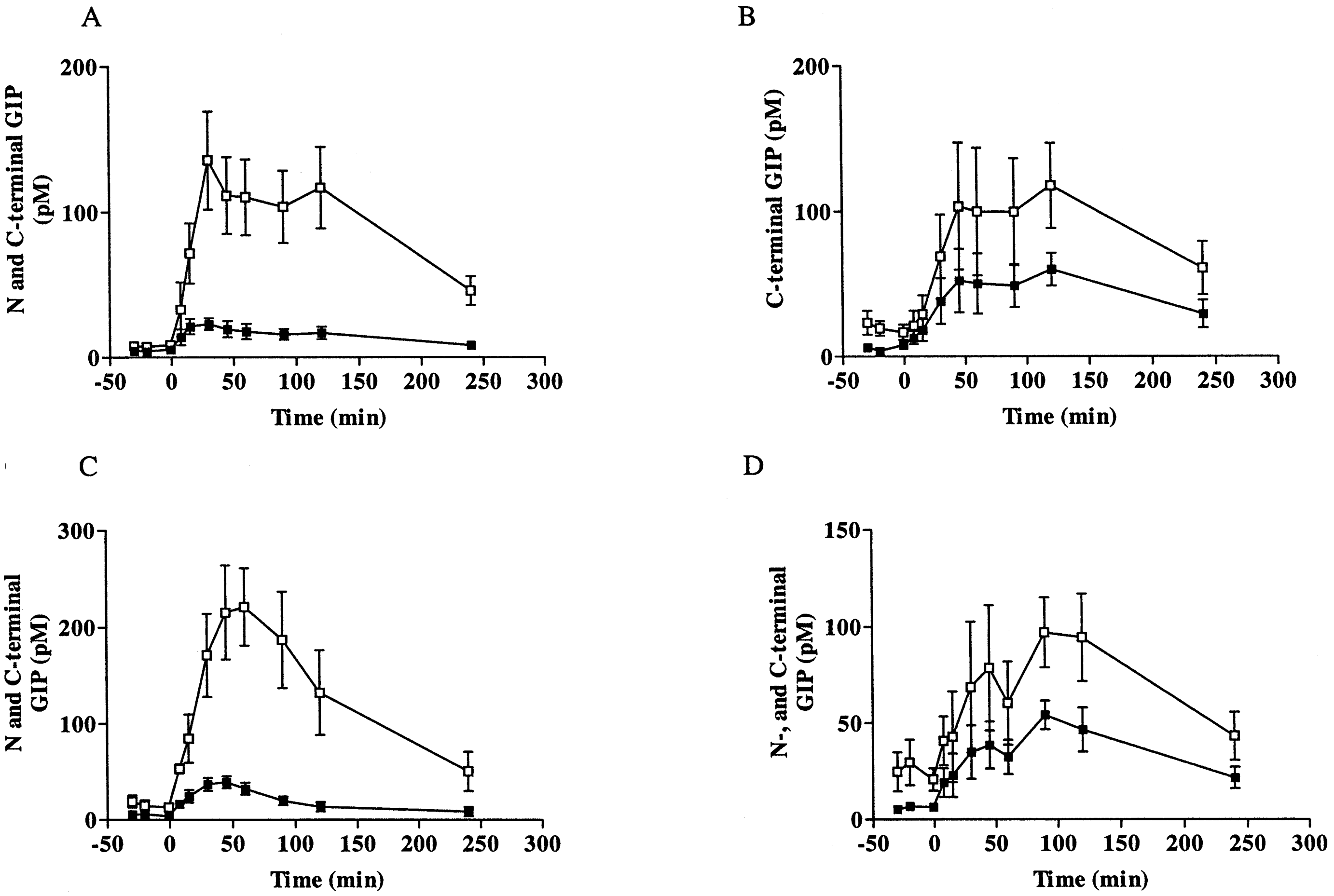

FIGURE 3
Plasma levels of intact (N-terminal) (■) and total (C-terminal) (□) GIP during OGTT in male Göttingen minipigs before (*A*, *B*) and after (*C*, *D*) dosing of NIA (67 mg/kg) and STZ (125 mg/kg). Animals are dosed with either vehicle (*A*, *C*) or VP (50 mg/kg) (*B*, *D*). Glucose (2 g/kg) ingested at $t = 0$ minutes, VP or vehicle dosed IV at $t = -15$ minutes. Data are presented as mean ± SEM, n = 6. Note different scales on Y-axis.

however, there was some variability of the effect of VP on DPPIV activity in the individual animals, especially before administration of NIA + STZ.

VP significantly improved glucose tolerance compared to vehicle treatment in animals previously dosed with NIA + STZ. Thus $AUC_{glucose}$ was decreased substantially, whereas no significant effect was seen in normal animals (Table 2). VP did not increase insulin levels during the OGTT in either normal animals or after administration of NIA + STZ, but rather, there was a tendency for insulin levels to be slightly lower after VP (although this failed to reach statistical significance) (Figure 1). In spite of the apparently lower insulin levels after VP, the insulinogenic index was increased by VP in normal animals (from 83 ± 42 to 192 ± 108; $P < .05$). This effect of VP was significantly larger ($P < .01$) than in animals dosed with NIA + STZ, where no significant insulinogenic effect of VP was seen (45 ± 23 in vehicle-treated animals versus 57 ± 29 in VP-dosed animals, NS).

DPPIV inhibition with VP did not affect the total amount of GIP (GIP $AUC_{C\text{-terminal}}$) released in response to the OGTT in normal animals, but surprisingly, in the group with reduced $\beta$-cell mass, total GIP levels showed a significant reduction (Table 3, Figure 3). In contrast, concentrations of intact GIP were elevated by VP in both normal animals and after NIA + STZ. This resulted in the proportion of GIP that remained intact and biologically active being significantly increased by VP in both groups.

Changes in GLP-1 concentrations were less obvious, due in part to the fact that data on total GLP-1 (amidated + glycine-extended) were only available for 3 out of the 6 animals. Total GLP-1 was not affected by VP in either normal pigs or after NIA + STZ (Table 3, Figure 2). Although they failed to

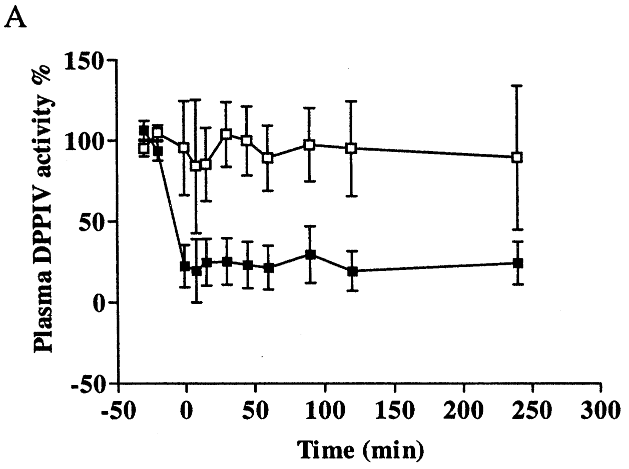

FIGURE 4
Plasma DPPIV activity during OGTT in male Göttingen minipigs before (*A*) and after (*B*) dosing of NIA (67 mg/kg) and STZ (125 mg/kg). Animals are dosed with either VP (50 mg/kg) (■) or vehicle (□). Glucose (2 g/kg) ingested at $t = 0$ minutes, VP or vehicle dosed IV at $t = -15$ minutes; n = 6. Data are presented as mean ± SD and are normalized according to baseline values.

reach statistical significance for the group as a whole, intact GLP-1 levels in individual animals showed a tendency to increase following VP administration, resulting in a trend toward an increased proportion of intact GLP-1.

### Proportion of Glycine-Extended C-Terminal GLP-1 Compared to Total C-Terminal GLP-1

Both amidated and glycine-extended GLP-1 are present in the pig. The use of the 2 different C-terminal assays, which are able to discriminate between the forms, revealed that glycine-extended GLP-1 is the predominant form in the pig, accounting for 74% ± 13% of total GLP-1 in the fasted state and 60% ± 4% of total GLP-1 $AUC_{C\text{-terminal}}$ in the postprandial state. Administration of NIA + STZ or VP did not change these proportions.

### DISCUSSION

DPPIV inhibitors are currently under investigation as new antidiabetic agents. They improve adverse glucose tolerance in rodents [1, 4, 56, 57, 63] and in type 2 diabetic patients in the early stages of the disease [2]. Their mechanism of action is believed to involve enhancement of endogenous incretins, and because of this, it is unknown whether they will also be effective when insulin secretory capacity is impaired. This study, therefore, aimed to examine the effect of DPPIV inhibition in a model of glucose intolerance due to reduced $\beta$-cell mass. The mild deterioration of glucose tolerance and insulin response during oral glucose following the modest (to 40%) $\beta$-cell reduction is in accord with human data showing that a more substantial $\beta$-cell reduction (to around 10% to 15%) is required before overt insulin-dependent diabetes develops [20, 32, 58].

DPPIV inhibitors structurally based upon the dipeptide product of DPPIV cleavage, such as VP, ile-thiazolidine, and NVPDPP728, are effective in vivo, inhibiting plasma DPPIV activity in pig, man, monkey, rat, and dog [4, 8, 12, 56, 67]. In this study, VP inhibited plasma DPPIV activity by over 90% during the OGTT, and was associated with increased levels of intact GIP. Total GIP levels (C-terminal assay) were unaffected by VP in normal animals, but the decrease seen in the glucose-intolerant group is in agreement with previous studies in normal dogs [12]. This was interpreted as reduced secretion of GIP [12], because the metabolic clearance rate of C-terminal GIP immunoreactivity is unchanged by DPPIV inhibition [7]. Enhanced intact GIP levels may feedback to inhibit further secretion, directly at the K cells or indirectly via another agent. In the latter case, insulin can inhibit GIP secretion [62], although in the present study, insulin levels were, if anything, lower after VP. GLP-1 concentrations were, surprisingly, apparently not affected by VP. However, this finding must be interpreted with some caution, because only 3 animals were available for analysis of total GLP-1 immunoreactivity, and, as noted earlier, there was a tendency for intact GLP-1 to make up a greater proportion of total GLP-1 following VP. Acute DPPIV inhibition prevents N-terminal degradation of exogenous GLP-1 [8], and increases levels of endogenous intact GLP-1 [1, 4, 12, 63]. After chronic DPPIV inhibition, intact GLP-1 levels increase in some [63], but not all [56], studies. The reason for these differences is unclear, and further studies designed to investigate the mechanisms regulating K- and L-cell activity are needed before the overall effect of DPPIV inhibition on incretin hormone secretion can be assessed.

In the present study, VP reduced the hyperglycemic response to oral glucose in glucose-intolerant animals. However, in contrast to results obtained after acute DPPIV inhibition in obese rodents [1, 4, 63], insulin levels were not increased, but were actually slightly lower than in vehicle-treated animals. This could reflect the integrated response of the pancreas to 2 simultaneous opposing stimuli, i.e., increased insulin secretion in response to elevated intact incretin hormone levels, together with a concomitant reduction of insulin secretion due to lower glucose levels. Indeed, reduced insulin responses to a glucose or meal challenge after GLP-1 infusion have been reported in some [22, 46, 49], but not all [65, 71, 74], studies in human subjects with type 2 diabetes, whereas decreased insulin secretory responses to GLP-1 have been observed in humans with impaired glucose tolerance compared to normal subjects [18]. However, other mechanisms may also contribute. In healthy subjects, a low GLP-1 infusion rate (0.4 pmol/kg/min) delayed gastric emptying, thereby reducing the glycemic excursion and, consequentially, insulin secretion, leading to the conclusion that the gastric emptying effects may outweigh the insulinotropic effects of GLP-1 [49]. Thus, even minor increases in endogenous intact GLP-1 in the present study may have influenced gastric emptying. However, mechanisms other than GLP-1–mediated effects must also be involved, because glucose tolerance is improved by DPPIV inhibition in mice with a specific deletion of the GLP-1 receptor [39]. GIP is one possibility, and the increased intact GIP levels found in the present study may have contributed to the effective uptake of glucose [44, 51, 52, 73]. After on-going DPPIV inhibition, improvements in glucose tolerance are associated with a reduction in insulinemia [2, 56, 63], which indirectly suggest an improvement in insulin sensitivity, and this is further supported by direct assessment of insulin sensitivity in glucose-intolerant rats after 12 weeks of DPPIV inhibition [57]. In the present study, the insulinogenic index (describing insulin levels relative to glycemia), used as a measure of $\beta$-cell sensitivity to prevailing glucose concentrations, was increased in normal animals, although this was not seen after NIA + STZ, possibly because of reduced residual insulin secretory capacity in these animals. Similar improvements in the insulinogenic index and insulin sensitivity are seen following long-term (6 weeks) continuous treatment with GLP-1 [74]. It is also likely that levels of other, as yet unidentified, substrates of DPPIV are enhanced, or that products of DPPIV action are reduced, by VP and may additionally contribute to the improvements in glucose tolerance.

In conclusion, these studies support the potential use of DP-PIV inhibitors as an approach to the treatment of adverse postprandial glucose where this is attributable to reduced $\beta$-cell mass. Taken together with observations that DPPIV inhibitors also reduce postprandial glycemia in hyperinsulinemic models of glucose intolerance [4, 56, 57], the data suggest that DPPIV inhibition may provide effective treatment of type 2 diabetes, irrespective of the etiology of the disease.

### REFERENCES

[1] Ahren, B., Holst, J. J., Martensson, H., and Balkan, B. (2000) Improved glucose tolerance and insulin secretion by inhibition of dipeptidyl peptidase IV in mice. *Eur. J. Pharmacol.,* **404,** 239–245.

[2] Ahren, B., Simonsson, E., Larsson, H., Landin-Olsson, M., Torgeirsson, H., Jansson, P. A., Sandqvist, M., Ovenholm, P., Efendic, S., Eriksson, J. W., Dickinson, S., and Holmes, D. (2002) Inhibition of dipeptidyl peptidase IV improves metabolic control over a 4-week study period in type 2 diabetes. *Diabetes Care,* **25,** 869–875.

[3] Andersen, L., Dinesen, B., Jørgensen, P. N., Poulsen, F., and Røder, M. E. (1993) Enzyme immunoassay for intact human insulin in serum or plasma. *Clin. Chem.,* **39,** 578–582.

[4] Balkan, B., Kwasnik, L., Miserendino, R., Holst, J. J., and Li, X. (1999) Inhibition of dipeptidyl peptidase IV with NVP-DPP728 increases plasma GLP-1 (7–36 amide) concentrations and improves glucose tolerance in obese Zucker rats. *Diabetologia,* **42,** 1324–1331.

[5] Bock, T., Svenstrup, K., Pakkenberg, B., and Buschard, K. (1999) Unbiased estimation of total beta-cell number and mean beta-cell volume in rodent pancreas. *APMIS,* **107,** 791–799.

[6] Brown, D. R., and Terris, J. M. (1996) *Swine in Physiological and Pathophysiological Research,* edited by Tumbleson, M. E., and Schook, L. B., pp. 5–6. New York, Plenum Press.

[7] Deacon, C. F., Danielson, P., Klarskov, L., Olesen, M., and Holst, J. J. (2001) Dipeptidyl peptidase IV inhibition reduces the degradation and clearance of GIP and potentiates its insulinotropic and antihyperglycemic effects in anesthetized pigs. *Diabetes,* **50,** 1588–1597.

[8] Deacon, C. F., Hughes, T. E., and Holst, J. J. (1998) Dipeptidyl peptidase IV inhibition potentiates the insulinotrophic effect of glucagon-like peptide 1 in the anesthetized pig. *Diabetes,* **47,** 764–769.

[9] Deacon, C. F., Johnsen, A. H., and Holst, J. J. (1995) Degradation of glucagon-like peptide-1 by human plasma in vitro yields an N-terminally truncated peptide that is a major endogenous metabolite in vivo. *J. Clin. Endocrinol. Metab.,* **80,** 952–957.

[10] Deacon, C. F., Nauck, M. A., Meier, J., Hucking, K., and Holst, J. J. (2000) Degradation of endogenous and exogenous gastric inhibitory polypeptide in healthy and in type 2 diabetic subjects as revealed using a new assay for the intact peptide. *J. Clin. Endocrinol. Metab.*, **85,** 3575–3581.

[11] Deacon, C. F., Nauck, M. A., Toft-Nielsen, M. B., Pridal, L., Willms, B., and Holst, J. J. (1995) Both subcutaneously and intravenously administered glucagon-like peptide I are rapidly degraded from the $NH_2$-terminus in type II diabetic patients and healthy subjects. *Diabetes,* **44,** 1126–1131.

[12] Deacon, C. F., Wamberg, S., Bie, P., Hughes, T. E., and Holst, J. J. (2002) Preservation of active incretin hormones by inhibition of dipeptidyl peptidase IV suppresses meal-induced incretin secretion in dogs. *J. Endocrinol.,* **172,** 355–362.

[13] Edwards, C. M., Todd, J. F., Mahmoudi, M., Wang, Z., Wang, R. M., Ghatei, M. A., and Bloom, S. R. (1999) Glucagon-like peptide 1 has a physiological role in the control of postprandial glucose in humans: Studies with the antagonist exendin 9–39. *Diabetes,* **48,** 86–93.

[14] Elahi, D., McAloon-Dyke, M., Fukagawa, N. K., Meneilly, G. S., Sclater, A. L., Minaker, K. L., Habener, J. F., and Andersen, D. K. (1994) The insulinotrophic actions of glucose-dependent insulinotropic polypeptide (GIP) and glucagon-like peptide 1 (7–37) in normal and diabetic subjects. *Regul. Pept.,* **51,** 63–74.

[15] Ellegaard, L., Jørgensen, K. D., Klastrup, S., Hansen, A. K., and Svendsen, O. (1995) Haematologic and clinical chemical values in 3 and 6 months old Göttingen minipigs. *Scand. J. Lab. Anim. Sci.,* **22,** 239–248.

[16] Fehmann, H. C., and Habener, J. F. (1992) Insulinotropic hormone glucagon-like peptide-I(7–37) stimulation of proinsulin gene expression and proinsulin biosynthesis in insulinoma beta TC-1 cells. *Endocrinology,* **130,** 159–166.

[17] Flint, A., Raben, A., Astrup, A., and Holst, J. J. (1998) Glucagon-like peptide 1 promotes satiety and suppresses energy intake in humans. *J. Clin. Invest.,* **101,** 515–520.

[18] Fritsche, A., Stefan, N., Hardt, E., Haring, H., and Stumvoll, M. (2000) Characterisation of beta-cell dysfunction of impaired glucose tolerance: Evidence for impairment of incretin-induced insulin secretion. *Diabetologia,* **43,** 852–858.

[19] Fujimori, K., Takahashi, A., Numata, H., and Takanaka, A. (1986) Drug metabolizing enzyme system of the Göttingen miniature pig. In: *Swine in Biomedical Research*, edited by Tumbleson, M. E., pp. 533–548. New York, Plenum Press.

[20] Gepts, W., and Lecompte, P. M. (1981) The pancreatic islets in diabetes. *Am. J. Med.,* **70,** 105–115.

[21] Gromada, J., Holst, J. J., and Rorsman, P. (1998) Cellular regulation of islet hormone secretion by the incretin hormone glucagon-like peptide 1. *Pflugers Arch.,* **435,** 583–594.

[22] Gutniak, M., Orskov, C., Holst, J. J., Ahren, B., and Efendic, S. (1992) Antidiabetogenic effect of glucagon-like peptide-1(7–36) amide in normal subjects and patients with diabetes mellitus. *N. Engl. J. Med.,* **326,** 1316–1322.

[23] Hansen, L., Hartmann, B., Bisgaard, T., Mineo, H., Jorgensen, P. N., and Holst, J. J. (2000) Somatostatin restrains the secretion of glucagon-like peptide-1 and -2 from isolated perfused porcine ileum. *Am. J. Physiol. Endocrinol. Metab.,* **278,** E1010–E1018.

[24] Holst, J. J., and Deacon, C. F. (1998) Inhibition of the activity of dipeptidyl-peptidase IV as a treatment for type 2 diabetes. *Diabetes,* **47,** 1663–1670.

[25] Holst, J. J., Gromada, J., and Nauck, M. A. (1997) The pathogenesis of NIDDM involves a defective expression of the GIP receptor. *Diabetologia,* **40,** 984–986.

[26] Jacobsson, L. (1986) Comparison of experimental hypercholesterolemia and atherosclerosis in Gottingen minipigs and Swedish domestic swine. *Atherosclerosis,* **59,** 205–213.

[27] Jacobsson, L. (1989) Comparison of experimental hypercholesterolemia and atherosclerosis in male and female minipigs of the Gottingen strain. *Artery,* **16,** 105–117.

[28] Johansen, T., Hansen, H. S., Richelsen, B., and Malmlöf, K. (2001) The obese Göttingen minipig as a model of the metabolic syndrome: Dietary effects on obesity, insulin sensitivity and growth hormone profile. *Comp. Med.,* **51,** 150–155.

[29] Jørgensen, K. D., Ellegaard, L., Klastrup, S., and Svendsen, O. (1998) Haematological and clinical chemical values in pregnant and juvenile Göttingen minipigs. *Scand. J. Lab. Anim. Sci.,* **25,** 181–190.

[30] Kieffer, T. J., McIntosh, C. H., and Pederson, R. A. (1995) Degradation of glucose-dependent insulinotropic polypeptide and truncated glucagon-like peptide 1 in vitro and in vivo by dipeptidyl peptidase IV. *Endocrinology,* **136,** 3585–3596.

[31] Kjems, L. L., Kirby, B. M., Welsh, E. M., Veldhuis, J. D., Straume, M., McIntyre, S. S., Yang, D. C., Lefebvre, P., and Butler, P. C. (2001) Decrease in beta-cell mass leads to impaired pulsatile insulin secretion, reduced postprandial hepatic insulin clearance, and relative hyperglucagonemia in the minipig. *Diabetes,* **50,** 2001–2012.

[32] Klöppel, G., Öhr, M., Habich, K., Oberholzer, M., and Heitz, P. U. (1985) Islet pathology and the pathogenesis of type 1 and type 2 diabetes mellitus revisited. *Surv. Synth. Pathol. Res.,* **4,** 110–125.

[33] Krarup, T., and Holst, J. J. (1984) The heterogeneity of gastric inhibitory polypeptide in porcine and human gastrointestinal mucosa evaluated with five different antisera. *Regul. Pept.,* **9,** 35–46.

[34] Krarup, T., Madsbad, S., Moody, A. J., Regeur, L., Holst, J. J., and Sestoft, L. (1983) Diminished immunoreactive gastric inhibitory polypeptide response to a meal in mewly diagnosed type I (insulin dependent) diabetics. *J. Clin. Endocrinol. Metab.,* **56,** 1306–1312.

[35] Larsen, M. O., Rolin, B., Wilken, M., Carr, R. D., Svendsen, O., and Bollen, P. (2001) Parameters of glucose and lipid metabolism in the male Gottingen minipig: Influence of age, body weight, and breeding family. *Comp. Med.,* **51,** 436–442.

[36] Larsen, M. O., Rolin, B., Wilken, M., and Svendsen, O. (2002) High fat high energy feeding impairs fasting glucose and increases fasting insulin levels in the Göttingen minipig—Results from a pilot study. *Ann. N. Y. Acad. Sci.,* **967,** 414–423.

[37] Larsen, M. O., Wilken, M., Gotfredsen, C. F., Carr, R. D., Svendsen, O., and Rolin, B. (2002) Mild streptozotocin diabetes in the Göttingen minipig. A novel model of moderate insulin deficiency and diabetes. *Am. J. Physiol. Endocrinol. Metab.,* **282,** E1342–E1351.

[38] Lewis, J. T., Dayanandan, B., Habener, J. F., and Kieffer, T. J. (2000) Glucose-dependent insulinotropic polypeptide confers early phase insulin release to oral glucose in rats: Demonstration by a receptor antagonist. *Endocrinology,* **141,** 3710–3716.

[39] Marguet, D., Baggio, L., Kobayashi, T., Bernard, A. M., Pierres, M., Nielsen, P. F., Ribel, U., Watanabe, T., Drucker, D. J., and Wagtmann, N. (2000) Enhanced insulin secretion and improved glucose tolerance in mice lacking CD26. *Proc. Natl. Acad. Sci. U.S.A.,* **97,** 6874–6879.

[40] Mayhew, T. M., and Gundersen, H. J. (1996) 'If you assume, you can make an ass out of u and me': A decade of the disector for stereological counting of particles in 3D space. *J. Anat.,* **188,** 1–15.

[41] Mentlein, R., Gallwitz, B., and Schmidt, W. E. (1993) Dipeptidyl-peptidase IV hydrolyses gastric inhibitory polypeptide, glucagon-like peptide-1(7–36) amide, peptide histidine methionine and is responsible for their degradation in human serum. *Eur. J. Biochem.,* **214,** 829–835.

[42] Miller, E. R., and Ullrey, D. E. (1987) The pig as a model for human nutrition. *Annu. Rev. Nutr.,* **7,** 361–382.

[43] Miyawaki, K., Yamada, Y., Yano, H., Niwa, H., Ban, N., Ihara, Y., Kubota, A., Fujimoto, S., Kajikawa, M., Kuroe, A., Tsuda, K., Hashimoto, H., Yamashita, T., Jomori, T., Tashiro, F., Miyazaki, J., and Seino, Y. (1999) Glucose intolerance caused by a defect in the entero-insular axis: A study in gastric inhibitory polypeptide receptor knockout mice. *Proc. Natl. Acad. Sci. U.S.A.,* **96,** 14843–14847.

[44] Morgan, L. M. (1998) The role of gastrointestinal hormones in carbohydrate and lipid metabolism and homeostasis: Effects of gastric inhibitory polypeptide and glucagon-like peptide-1. *Biochem. Soc. Trans.,* **26,** 216–222.

[45] Naslund, E., Gutniak, M., Skogar, S., Rossner, S., and Hellstrom, P. M. (1998) Glucagon-like peptide 1 increases the period of postprandial satiety and slows gastric emptying in obese men. *Am. J. Clin. Nutr.,* **68,** 525–530.

[46] Nathan, D. M., Schreiber, E., Fogel, H., Mojsov, S., and Habener, J. F. (1992) Insulinotropic action of glucagonlike peptide-I-(7–37) in diabetic and nondiabetic subjects. *Diabetes Care,* **15,** 270–276.

[47] Nauck, M. A., Heimesaat, M. H., Ørskov, C., Holst, J. J., Ebert, R., and Creutzfeldt, W. (1993) Preserved incretin activity of glucagon-like peptide 1(7–36 amide) but not synthetic human gastric inhibitory polypeptide in patients with type-2 diabetes mellitus. *J. Clin. Invest.,* **91,** 301–307.

[48] Nauck, M. A., Kleine, N., Orskov, C., Holst, J. J., Willms, B., and Creutzfeldt, W. (1993) Normalization of fasting hyperglycaemia by exogenous glucagon-like peptide 1 (7–36 amide) in type 2 (non-insulin-dependent) diabetic patients. *Diabetologia,* **36,** 741–744.

[49] Nauck, M. A., Niedereichholz, U., Ettler, R., Holst, J. J., Orskov, C., Ritzel, R., and Schmiegel, W. H. (1997) Glucagon-like peptide 1 inhibition of gastric emptying outweighs its insulinotropic effects in healthy humans. *Am. J. Physiol.,* **273,** E981–E988.

[50] Neubert, K., Born, I., Faust, J., Heins, J., Barth, A., Demuth, H. U., Rahfeld, J. U., and Steinmetzer, T. (1983) Verfahren zur herstellung neuer inhibitoren der dipeptidyl peptidase IV. German Patent Application number DD 296 075 A5.

[51] O'Harte, F. P. M., Gray, A. M., and Flatt, P. R. (1998) Gastric inhibitory polypeptide and effects of glycation on glucose transport and metabolism in isolated mouse abdominal muscle. *J. Endocrinol.,* **156,** 237–243.

[52] Oben, J., Morgan, L., Fletcher, J., and Marks, V. (1991) Effect of the entero-pancreatic hormones, gastric inhibitory polypeptide and glucagon-like polypeptide-1(7–36) amide, on fatty acid synthesis in explants of rat adipose tissue. *J. Endocrinol.,* **130,** 267–272.

[53] Orskov, C., Holst, J. J., and Nielsen, O. V. (1988) Effect of truncated glucagon-like peptide-1 proglucagon-(78–107) amide on endocrine secretion from pig pancreas, antrum, and nonantral stomach. *Endocrinology,* **123,** 2009–2013.

[54] Orskov, C., Jeppesen, J., Madsbad, S., and Holst, J. J. (1991) Proglucagon products in plasma of noninsulin-dependent diabetics and nondiabetic controls in the fasting state and after oral glucose and intravenous arginine. *J. Clin. Invest.,* **87,** 415–423.

[55] Pauly, R. P., Rosche, F., Wermann, M., McIntosh, C. H., Pederson, R. A., and Demuth, H. U. (1996) Investigation of glucose-dependent insulinotropic polypeptide (1–42) and glucagon-like peptide-1-(7–36) degradation in vitro by dipeptidyl peptidase IV using matrix-assisted laser desorption/ionization-time of flight mass spectrometry. A novel kinetic approach. *J. Biol. Chem.,* **271**, 23222–23229.

[56] Pospisilik, J. A., Stafford, S. G., Demuth, H. U., Brownsey, R., Parkhouse, W., Finegood, D. T., McIntosh, C. H., and Pederson, R. A. (2002) Long-term treatment with the dipeptidyl peptidase IV inhibitor P32/98 causes sustained improvements in glucose tolerance, insulin sensitivity, hyperinsulinemia, and beta-cell glucose responsiveness in VDF (fa/fa) Zucker rats. *Diabetes,* **51,** 943–950.

[57] Pospisilik, J. A., Stafford, S. G., Demuth, H. U., McIntosh, C. H., and Pederson, R. A. (2002) Long-term treatment with dipeptidyl peptidase IV inhibitor improves hepatic and peripheral insulin sensitivity in the VDF Zucker rat: A euglycemic-hyperinsulinemic clamp study. *Diabetes,* **51,** 2677–2683.

[58] Saito, K., Yaginuma, N., and Takahashi, T. (1979) Differential volumetry of A, B and D cells in the pancreatic islets of diabetic and nondiabetic subjects. *Tohoku. J. Exp. Med.,* **129,** 273–283.

[59] Schirra, J., Sturm, K., Leicht, P., Arnold, R., Goke, B., and Katschinski, M. (1998) Exendin(9–39) amide is an antagonist of glucagon-like peptide-1(7–36) amide in humans. *J. Clin. Invest.,* **101,** 1421–1430.

[60] Scrocchi, L. A., Brown, T. J., MaClusky, N., Brubaker, P. L., Auerbach, A. B., Joyner, A. L., and Drucker, D. J. (1996) Glucose intolerance but normal satiety in mice with a null mutation in the glucagon-like peptide 1 receptor gene. *Nat. Med.,* **2,** 1254–1258.

[61] Starich, G. H., Bar, R. S., and Mazzaferri, E. L. (1985) GIP increases insulin receptor affinity and cellular sensitivity in adipocytes. *Am. J. Physiol.,* **249,** E603–E607.

[62] Stockmann, F., Ebert, R., and Creutzfeldt, W. (1984) Preceding hyperinsulinemia prevents demonstration of insulin effect on fat-induced gastric inhibitory polypeptide (GIP). *Diabetes,* **33,** 580–585.

[63] Sudre, B., Broqua, P., White, R. B., Ashworth, D., Evans, D. M., Haigh, R., Junien, J. L., and Aubert, M. L. (2002) Chronic inhibition of circulating dipeptidyl peptidase IV by FE 999011 delays the occurrence of diabetes in male Zucker diabetic fatty rats. *Diabetes,* **51,** 1461–1469.

[64] Swindle, M. M., and Smith, A. C. (1998) Comparative anatomy and physiology of the pig. *Scand. J. Lab. Anim. Sci.,* **25,** 11–21.

[65] Toft-Nielsen, M. B., Madsbad, S., and Holst, J. J. (1999) Continuous subcutaneous infusion of glucagon-like peptide 1 lowers plasma glucose and reduces appetite in type 2 diabetic patients. *Diabetes Care,* **22,** 1137–1143.

[66] Tseng, C. C., Kieffer, T. J., Jarboe, L. A., Usdin, T. B., and Wolfe, M. M. (1996) Postprandial stimulation of insulin release by glucose-dependent insulinotropic polypeptide (GIP). Effect of a specific glucose-dependent insulinotropic polypeptide receptor antagonist in the rat. *J. Clin. Invest.,* **98,** 2440–2445.

[67] Villhauer, E. B., Brinkman, J. A., Naderi, G. B., Dunning, B. E., Mangold, B. L., Mone, M. D., Russell, M. E., Weldon, S. C., and Hughes, T. E. (2002) 1-[2-[(5-Cyanopyridin-2-yl)amino]-ethylamino]acetyl-2-(*S*)-pyrrolidine-carbonitrile: A potent, selective, and orally bioavailable dipeptidyl peptidase IV inhibitor with antihyperglycemic properties. *J. Med. Chem.,* **45,** 2362–2365.

[68] Wang, Y., Perfetti, R., Greig, N. H., Holloway, H. W., DeOre, K. A., Montrose-Rafizadeh, C., Elahi, D., and Egan, J. M. (1997) Glucagon-like peptide-1 can reverse the age-related decline in glucose tolerance in rats. *J. Clin. Invest.,* **99,** 2883–2889.

[69] Weir, G. C., Mojsov, S., Hendrick, G. K., and Habener, J. F. (1989) Glucagonlike peptide I (7–37) actions on endocrine pancreas. *Diabetes,* **38,** 338–342.

[70] Wettergren, A., Schjoldager, B., Mortensen, P. E., Myhre, J., Christiansen, J., and Holst, J. J. (1993) Truncated GLP-1 (proglucagon 78–107-amide) inhibits gastric and pancreatic functions in man. *Dig. Dis. Sci.,* **38,** 665–673.

[71] Willms, B., Werner, J., Holst, J. J., Orskov, C., Creutzfeldt, W., and Nauck, M. A. (1996) Gastric emptying, glucose responses, and insulin secretion after a liquid test meal: Effects of exogenous glucagon-like peptide-1 (GLP-1)-(7–36) amide in type 2 (noninsulin-dependent) diabetic patients. *J. Clin. Endocrinol. Metab.,* **81,** 327–332.

[72] Wishart, J. M., Horowitz, M., Morris, H. A., Jones, K. L., and Nauck, M. A. (1998) Relation between gastric emptying of glucose and plasma concentrations of glucagon-like peptide-1. *Peptides,* **19,** 1049–1053.

[73] Yip, R. G. C., and Wolfe, M. M. (2000) GIP biology and fat metabolism. *Life Sci.,* **66,** 91–103.

[74] Zander, M., Madsbad, S., Madsen, J. L., and Holst, J. J. (2002) Effect of 6-week course of glucagon-like peptide 1 on glycaemic control, insulin sensitivity, and beta-cell function in type 2 diabetes: A parallel-group study. *Lancet,* **359,** 824–830.

[75] Ørskov, C., Rabenhøj, L., Wettergren, A., Kofod, H., and Holst, J. J. (1994) Tissue and plasma concentrations of amidated glycine-extended glucagon-like peptide-1 in humans. *Diabetes,* **43,** 535–539.

